# Supplementary figures and images for: Temporal Trends in Treatment and Outcomes of Endometrial Carcinoma in the United States, 2005–2020
Source: Cancers (Basel). 2024 Mar 26;16(7):1282. doi: 10.3390/cancers16071282 (PMC11011139; doi:10.3390/cancers16071282)

## Supplemental Material

**Figure S1:** Trends in the sequencing of systemic therapy (2006–2020).

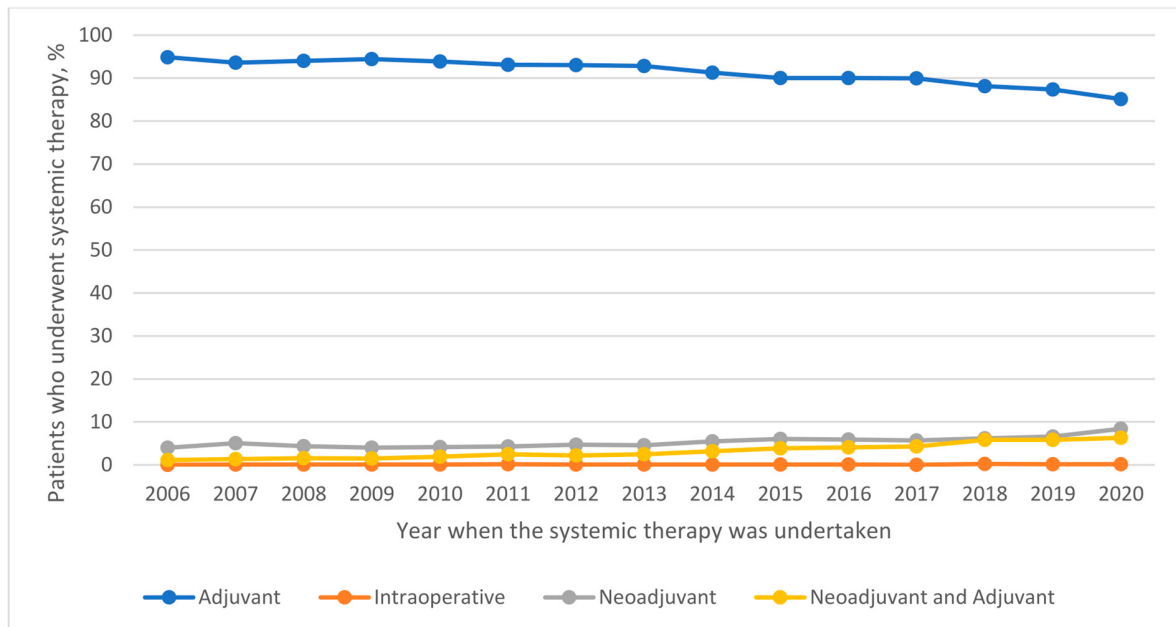

Supplement: Supplementary file 1 [file cancers-16-01282-s001.zip › cancers-2891537-supplementary.pdf]
